# Identification of the Short Neuropeptide F and Short Neuropeptide F Receptor Genes and Their Roles of Food Intake in *Dendroctonus armandi*

**DOI:** 10.3390/insects12090844

**Published:** 2021-09-19

**Authors:** Bin Liu, Danyang Fu, Hang Ning, Ming Tang, Hui Chen

**Affiliations:** 1 College of Forestry, Northwest A&F University, Xianyang 712100, China; 18829355106@163.com (B.L.); fudanyang@nwsuaf.edu.cn (D.F.); ninghang92@gmail.com (H.N.); 2 State Key Laboratory for Conservation and Utilization of Subtropical Agro-Bioresources, Guangdong Key Laboratory for Innovative Development and Utilization of Forest Plant Germplasm, College of Forestry and Landscape Architecture, South China Agricultural University, Guangzhou 510642, China

**Keywords:** *Dendroctonus armandi*, short neuropeptide F, short neuropeptide F receptor, food intake, molecular target, RNA interference

## Abstract

**Simple Summary:**

The Chinese white pine beetle, *Dendroctonus armandi*, is a destructive pest of coniferous forests in the middle Qinling Mountains of China. The short neuropeptide F (*sNPF*) and short NPF receptor (s*NPFR*) genes have important roles in a broad range of physiological and behavioral processes. However, the function of sNPF signaling pathway in regulating the food intake of *Dendroctonus armandi* has been unclear. In this study, we cloned and characterized cDNAs encoding sNPF and sNPF receptor in *D. armandi* and analyzed their spatiotemporal expression patterns, as well as changes in expression after starvation. Addtionally, sNPF and sNPFR knockdown in beetles using RNA interference significantly increased mortality and reduced their food intake and body weight through changes of a biosynthesis and metabolism pathway. From these results, we conclude that sNPF signaling pathway is important in the feeding control of *D. armandi.*

**Abstract:**

The short neuropeptide F (sNPF) is an essential signaling molecule that is evolutionarily conserved and involved in a broad range of physiological functions in the invertebrates, by interacting with sNPF receptors, which belong to G protein-coupled receptors (GPCR). However, the function of sNPF in regulating the food intake of *Dendroctonus armandi* has been unclear. In this study, we cloned and characterized cDNAs encoding sNPF and sNPF receptor in the *D. armandi* and made bioinformatics predictions on the deduced amino acid sequences. They had a high degree of similarity to that of *Dendroctonus ponderosa.* Quantitative real-time reverse transcription PCR (qRT-PCR) revealed that the transcript levels of both sNPF and sNPFR varied across developmental stages and body parts. In addition, the sNPF and sNPFR expression levels were upregulated in starved beetles, and the expression levels recovered after re-feeding. Furthermore, RNAi knockdown by the injection of sNPF and sNPFR dsRNA into beetles significantly increased mortality and reduced their food intake and body weight, and also caused decrease of glycogen and free fatty acid and increase of trehalose. These results indicate that sNPF signaling pathway plays an important role in the regulation of food intake and provides a potential molecular target for the eco-friendly control strategies of this pest.

## 1. Introduction

Neuropeptides are essential signaling molecules that are evolutionarily conserved and involved in a broad range of physiological functions. One type of these insect neuropeptides is the short neuropeptide F (sNPF), which is characterized by the xPxLRLRFamide sequence at the carboxy terminus [1]. Although peptides of the sNPF family serve as neuro-modulators with a crucial role in a wide range of physiological processes, such as hormone release, circadian rhythm, sleep homeostasis, stress resistance and metabolism, learning and memory, locomotion behavior, and olfactory sensitivity [2,3,4,5,6,7,8,9,10,11], their primary function appears to be the regulation of feeding and foraging behavior.

The first documented role of these effects on the regulation of feeding behavior was observed in *Drosophila melanogaster*, wherein overexpression of sNPF caused an increase of food uptake in both larvae and adult flies, leading to the formation of larger flies. Conversely, knocking down the sNPF precursor showed the opposite phenotype. Similarly, when the *Drosophila* sNPF receptor was overexpressed, this led to an augmentation in both body size and food uptake, while RNA interference (RNAi) mediated knockdown of the receptor resulted in smaller animals [12].

This positive correlation between sNPF signaling and feeding behavior was also demonstrated in several other insect species. In the bird cherry-oat aphid, *Rhopalosiphum padi*, starvation caused an increase in sNPF and sNPFR expression, whereas subsequent refeeding caused a tendency toward downregulation of these genes. sNPF knockdown also notably decreased adults’ food intake [13]. Moreover, when sNPF and sNPFR expression levels were observed in the honey bee, *Apis mellifera*, they were found to be higher in foragers that engage in food-searching behavior compared to nurses that provide brood care [14]. Furthermore, injection of sNPF-2 in the silk moth, *Bombyx mori*, significantly reduced latency to feeding, making the animals more prone to feed [15]. Although sNPF signaling seems to cause a positive effect on feeding in the above species, it is not the case for all insect species. When AedaeHP-I (sNPF-like peptide) was injected in the female yellow fever mosquito, *Aedes aegypti*, host-seeking behavior was significantly inhibited [16]. In the fire ant, *Solenopsis invicta*, the expression levels of the sNPF receptor were higher in controls compared to starved animals [17]. In *B. mori*, the expression levels of sNPF-1 and sNPF-2, as well as the sNPF receptor, decreased after starvation [18]. Additionally, in the American cockroach, *Periplaneta americana*, sNPF inhibited the digestive activity of the midgut when they were starved [19]. The expression level of the sNPF precursor was significantly lower in starved animals than that in non-starved controls in the desert locust, *Schistocerca gregaria* [20].

Chinese white pine beetle, *Dendroctonus armandi* Tsai and Li (Coleoptera: Curculionidae: Scolytinae), is a particularly destructive pest of coniferous forests in the middle Qinling and Bashan Mountains of China, not only invading healthy Chinese white pine, *Pinus armandii*, that are more than 30 years old, but also attracting other pests to the host trees, damaging the forest ecosystem and causing serious economic losses [21]. sNPFs have been reported in several insect species, as well as in beetles, and the first sNPF was identified in the brain of the Colorado potato beetle, *Leptinotarsa decemlineata* [22]. sNPF levels decreased in diapausing individuals, indicating their role in growth and development [23]. Short neuropeptide F signaling also inhibited the oviduct muscle contractility, stimulated the contractions of the ejaculatory duct, and regulated the functioning of the male reproductive system in yellow mealworm, *Tenebrio molitor* [24,25,26]. However, there are no reports on the functional roles of sNPF in *D. armandi.* In this study, we identified the full-length s*NPF* and s*NPFR* cDNAs from *D. armandi and* performed gene expression analysis and RNA interference experiments. These results provide an important step forward to the improvement of eco-friendly pest control strategies of bark beetles.

## 2. Materials and Methods

### 2.1. Insect Sample Preparation

Chinese white pine beetles were collected from infested *Pinus armandii* at the Huoditang Experimental Forest Station, which is on the southern slopes of the mid Qinling Mountains, Shaanxi, China (33°18′ N, 108°21′ E). Then, they were moved to the laboratory in plastic containers with moist paper and immediately stored at –80 °C until analysis. Logging slash of infested *P. armandi* was transported from the sample plot to a laboratory, where emerged adult beetles and larvae were collected and then reared in glass dishes (90 × 15 mm^2^) using the meridic diet described by a previous report [27]. All conditions were controlled by an artificial climate cabinet under certain conditions of 25 ± 1 °C, 70% relative humidity (RH) and in the dark. Adults were sexed by the external genitalia and male-specific auditory cues [28,29].

### 2.2. Total RNA Isolation and cDNA Synthesis

Total RNA was isolated from three development stages (larvae, pupae, and adults from both sexes) according to the UNlQ-10 Column Trizol Total RNA Isolation Kit (Sangon Biotech, Shanghai, China) on the basis of the manufacturer’s protocol. Beetles of each stage were used for total RNA isolation with complete bodies. The integrity of RNA was checked on 1.0% agarose gels electrophoresis and quantified by Nano Drop 2000 (Thermo Fisher Scientific, Inc., Pittsburgh, PA, USA). The purity was estimated by the average value of the A260/A280. Total mixed RNA for cDNA synthesis were carried out by the Fast King RT reagent Kit with gDNA Eraser (Tiangen, China) in a 20 µL reaction mixture containing 800 ng of total RNA, according to the manufacturer’s protocol, and then stored at −20 °C until analyzed.

### 2.3. Identification of D. armandi NPF and NPFR Genes

#### 2.3.1. cDNA Amplification and Cloning

The synthesized cDNA obtained from the samples was used as a template for the PCR. Each pair of specific primer (Table S1) for s*NPF* and s*NPFR* was designed by Primer Premier 5.0, on the basis of the unpublished transcriptome database [28]. All the PCR amplification were performed with a C1000 thermocycler (Bio-Rad Laboratories, Hercules, CA, USA) in a final mixture volume of 20 μL, containing 1 μL cDNA (1:4 dilution), 0.25 μL of each primer, 10 μL 2 × EcoTaq PCR SuperMix (TransGen, Beijing, China), and sterile water. The reaction conditions were as follows: 3 min at 95 °C, 30 cycles of 30 s at 95 °C, 30 s at 48–60 °C, 1 min at 72 °C, and a final extension of 10 min at 72 °C. PCR products were checked using a 1 × 4S Red Plus Nucleic Acid Stain (Sangong, Shanghai, China) in 1% agarose gels. The obtained PCR products were purified using a Gel Purification Kit (Omega BioTek, Norcross, GA, USA) and ligated into pMDTM 18-T vector (TaKaRa, Dalian, China), then transformed into DH5α chemically competent *Escherichia coli* cells (TransGen, Beijing, China). Following overnight culture in an incubator, white colonies were selected on Amp/LB/X-Gal/IPTG plates, and three positive colonies with the putative inserts using vector-specific primers (M13-F and M13-R) were sequenced by Augct Biotech, Beijing, China. Sequences were manually edited with the DNAMAN software to obtain inserts, which were blasted against the NCBI database.

#### 2.3.2. 3′ RACE and Cloning of Full-Length cDNA

Gene-specific primers for 5′ and 3′ RACE (Table S1) were designed according to the obtained sequence fragments. The 5′ and 3′ regions of the target gene were amplified using a SMARTerTM RACE cDNA amplification kit (Clontech, Mountain View, CA, USA) following the manufacturer’s instructions. Touchdown PCR was performed as follows: 95 °C for 2 min; 5 cycles of 94 °C for 30 s and 72 °C for 2 min; 5 cycles of 94 °C for 30 s, 65 °C for 30 s, and 72 °C for 90 s; 30 cycles of 94 °C for 30 s, 55 °C for 30 s, and 72 °C for 90 s; and a final incubation at 72 °C for 10 min. The amplified products were cloned, sequenced, and blasted as followed previous descriptions (see Section 2.3.1). To obtain the full-length gene, we designed specific primers containing the putative initiation and terminator codons (Table S1).

#### 2.3.3. Analysis of cDNA Sequences

The two cDNA sequences obtained were deposited in the GenBank, and accession numbers are listed in Table 1. The open reading frames (ORFs) of full-length cDNA sequences were obtained using ORF Finder (https://www.ncbi.nlm.nih.gov/orffinder/, accessed on 12 September 2020). Multiple sequence alignment of sequences was carried out in *DNAMAN6.0*. Molecular mass (kDa) and isoelectric points were determined in the ProtParam tool [30]. The putative signal peptide was predicted using Signal P 4.1 Server (http://www.cbs.dtu.dk/services/SignalP/, accessed on 12 September 2020). All putative *D. armandi* sNPF and sNPFR proteins were predicted for subcellular localization using the TARGETP tool (http://www.cbs.dtu.dk/services/TargetP/, accessed on 12 September 2020) [31] with the default parameters. TMHMM v. 2.0 (http://www.cbs.dtu.dk/services/TMHMM/, accessed on 12 September 2020) was used to visually display predictions of topological and transmembrane domains. The phylogenetic trees were constructed by software MEGA 6.0 [32] using the maximum likelihood method with a Whelan and Goldman (WAG) model and a gamma parameter value. The support for each node of bootstrap was estimated by a bootstrap program after 500 replicates. The data sets of sNPF and sNPFR sequences were chosen from other insect species.

### 2.4. Expression Patterns of sNPF and sNPFR Genes in Different Life Stages, Body Parts, and Treatments

#### 2.4.1. Insects Sampling and Treatments for RT-qPCR

*D. armandi* larvae were separated into two sub-stages: larvae (eating on host phloem for development) and mature larvae (cease feeding). Pupae were also separated into two sub-stages: early pupae (newly metamorphosed from larvae) and late pupae (close to becoming adults). We separated the adults into three substages: teneral adults (light body color), emerged adults, and feeding adults (invading a new host) [28]. At least 6 beetles from each stage were collected separately. Each beetle substage was killed with liquid nitrogen immediately after collection. We dissected heads, thoracic ganglions, foreguts, midguts, hindguts, pheromone glands, and body fat of emerged adults, and heads, thoracic ganglions, foreguts, midguts, hindguts, and fat body of larvae for analysis of expression in body parts. At least 12 beetles from each part were collected separately. Three biological replicates were collected, and each was analyzed in triplicate.

The males and females of emerged adults were divided into eight groups, and larvae were divided into seven groups. One group of collected insects, 0 h of feeding as control, were killed at time zero. Each of the other groups of emerged adults was immediately placed in glass dishes (90 × 15 mm^2^) with normal food for 24 and 48 h in the artificial climate cabinet. After 48 h of normal feeding, the insects were reared without food and starved for 72 h. Then, the live adults were re-fed for 24 h and larvae were investigated in the subsequent starvation treatment for 24 and 48 h. All of the beetles were checked for viability under a dissecting microscope, and 10 individuals were chosen from each sample and time point for use in real-time PCR analyses.

#### 2.4.2. RT-qPCR

The frozen samples were used for RNA isolation and cDNA first-strand synthesis followed previous descriptions (see Section 2.2). All samples were placed in the CFX-96 real-time PCR Detection System (Bio-Rad, CA, USA) for RTqPCR. The CYP4G55 (accession number: JQ855658.1) and β-actin (accession number: KJ507199.1) sequences of *D*. *armandi* were used as the internal control [33,34]. RT-qPCR primers were designed in Primer Premier 5.0 according to the obtained nucleotide sequences (Table S1). The specificity of qPCR primers was confirmed by the melting curve. To estimate the qPCR efficiency and validation for each gene, we performed a linear regression analysis between the mean values of quantification cycles of different dilutions (1.0, 10^−1^, 10^−2^, 10^−3^, 10^−4^, and 10^−5^) of cDNAs and their initial concentration. These dilutions were created from a cDNA pool, and 5 μL of each dilution was used as the qPCR template. PCR was performed 3 times for each gene, and its efficiency was estimated using the following formula: Efficiency = (10^−1/slope^ − 1) × 100, where the efficiency value was 100 ± 5%. The PCR validation was estimated directly from R^2^ values, which were *>*0.95. Three biological and three technical replicates were included to ensure reproducibility. The relative quantification of the expression levels was analyzed using the 2^−ΔΔCt^ method [35,36].

### 2.5. RNA Interference

#### 2.5.1. The dsRNA Synthesis and Injection

The T7 Ribo-MAXTM Express RNAi System (Promega, Madison, MI, USA) was used for the synthesis of dssNPF (196 bp), dssNPFR (354 bp), and dsGFP(395). RNAi primers (Table S1) were designed according to the obtained sequences. To prevent off-target effects, we chose specific target fragments to avoid any overlap with other genes, and the sequence specificity of target fragments was tested via NCBI BLAST. The final dsRNA products were diluted to 1000 ng/µL with diethyl pyrocarbonate (DEPC)-treated water, then stored at –80 °C and used within 6 months. Before injection, D. armandi were placed in an ice bath for 10 min. The beetles were immobilized on an agarose plate using manual forceps [37]. Afterward, each of the D. armandi emerged adults was microinjected with 0.2 µL dsRNA solution, and for larvae the amount was 0.l µL. Non-injected beetles and beetles injected with dsGFP were used as controls in all experiments. Then, they were transferred into an artificial climate cabinet for starvation or feeding. Each treatment group contained 40 individuals, and 6 individuals of each treatment were collected at 72 h after injection and then stored at −80 °C until qRT-PCR. Each treatment group contained three biological replicates.

#### 2.5.2. Survival Test and Body Weight Measurement

Adults and larvae mortality were observed under the different injected time points (12, 24, 36, 48, 60, 72 h) and control conditions. The beetles that did not move were considered to be dead when left at room temperature for 1 h [37]. After injection at 72 h, the body weight of each alive sample was immediately measured using an electronic balance. (d = 0.0001 g, AL204; Mettler-Toledo Ltd., Tianjin, China)

#### 2.5.3. Determination of Glycogen, Free Fatty Acid, Trehalose

For emerged adults and larvae at 72 h after injection, we measured three physiological indices in each group, namely, the content of glycogen, trehalose, and free fatty acid, using appropriate biochemical methods. Three biological replicates (six beetles for one replicate) were performed for each measurement. In addition, whole-body homogenates of each group were used to extract glycogen, trehalose, and free fatty acid. These three substances content levels were detected as previously described [27].

### 2.6. Statistical Analysis

All the statistical data were analyzed with SPSS Statistics 19.0 (IBM, Chicago, IL, USA). Post hoc Tukey tests were used to perform the significance of the difference through one-way ANOVA. Student’s *t*-test was performed with the two-sample analyses. Prism 6.0 (GraphPad Software, CA, USA) was used to construct the graphs.

## 3. Results

### 3.1. Sequence Characteristics and Bioinformatics Analysis

We successfully cloned cDNAs encoding sNPF and sNRFR from *D. armandi* with full coding region sequences, as well as sNPF cDNA encoding 102 amino acids, with molecular weights (MW) and isoelectric points (IP) of 11.48 kDa and 8.06, respectively (Table 1). On the other hand, sNPFR encoded 428 amino acids with molecular weights (MW) and isoelectric points (IP) of 49.27 kDa and 8.20, respectively (Table 1). The cellular localization of these proteins shows that sNPF is a secretory protein and that sNPFR is a membrane protein (Table 1).

**Figure 1:**
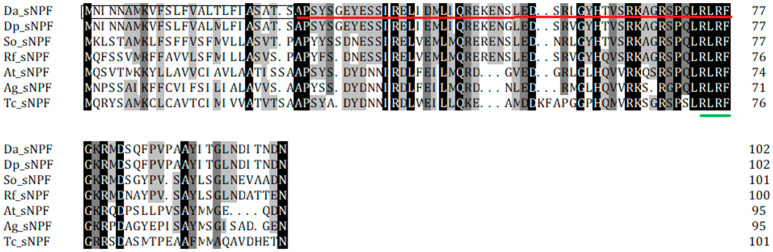
Deduced amino acid sequence of D. armandi sNPF and comparison of the amino acid sequence of the sNPFs with those of other species. They include Dendroctonus ponderosae (Dp sNPF, XP_019767920.1), *Sitophilus oryzae* (So sNPF, XP_030755875.1), *Rhynchophorus ferrugineus* (Rf sNPF, QGA72576.1), *Aethina tumida* (At sNPF, XP_019874673.1), *Anoplophora glabripennis* (Ag sNPF, XP_018580111.1), and *Tribolium castaneum* (Tc sNPF, XP_008198705.1). The putative signal region is indicated by black box, and the mature peptide is underlined by a solid red line. The typical *C*-terminal consensus sequence of sNPFs in Coleoptera is underlined by a solid green line. Identical amino acid residues in all proteins are shown in black; gray parts indicate similar amino acids.

**Figure 2:**
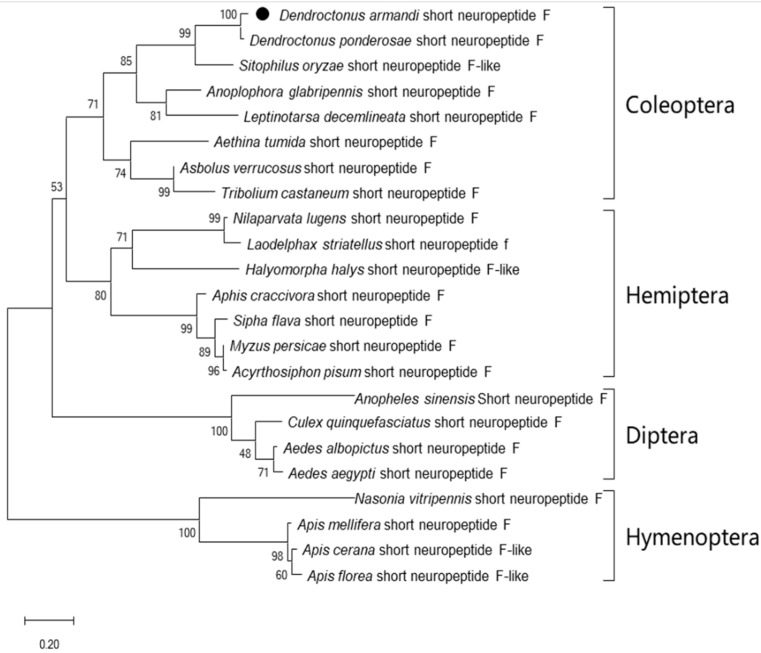
Phylogenetic trees based on amino acid sequences of sNPF from other insect species. They include Dendroctonus ponderosae (XP_019767920.1), *Sitophilus oryzae* (XP_030755875.1), *Rhynchophorus ferrugineus* (QGA72576.1), *Anoplophora glabripennis* (XP_018580111.1), *Leptinotarsa decemlineata* (XP_023021371.1), *Aethina tumida* (XP_019874673.1), *Asbolus verrucosus* (RZC41868.1), *Tribolium castaneum* (XP_008198705.1), *Nilaparvata lugens* (XP_022184541.1), *Laodelphax striatellus* (AXF48203.1), *Sipha flava* (XP_025411079.1), *Myzus persicae* (XP_022169760.1), *Acyrthosiphon pisum* (XP_003247250.1), *Anopheles sinensis* (KFB48121.1), *Culex quinquefasciatus* (AVR59281.1), *Aedes albopictus* (XP_029728321.1), *Aedes aegypti* (XP_021700214.1), *Nasonia vitripennis* (XP_008205837.1), *Apis mellifera* (XP_003250155.1), *Apis cerana* (XP_016914163.1), and *Apis florea* (XP_031773012.1). The phylogenetic tree constructed by the maximum likelihood method using the amino acidic substitution model WAG++G+I+F122 in MEGA5.0. Bootstrap values after 500 pseudo-replicates are shown at nodes. The bootstrap values (in %) are given at each branch point. The black dot indicates D. armandi sNPF.

The sNPF precursor had an N-terminal signal peptide of 25 amino acids and this was followed by the mature sNPF (Figure 1). The sNPF precursor contained the -RLRF sequence, which was followed by an amidation site (G) and a dibasic cleavage site (KR) (Figure 1). The protein sequence of sNPFR was predicted to have seven transmembrane domains and displayed typical hallmarks of the rhodopsin-like GPCR family (Figure S1). Further deduced amino acid sequence identities demonstrated that sNPF and sNPFR of D. armandi were highly similar with that of fellow Coleoptera member *D. ponderosa* (Table 2). The phylogenetic tree of sNPF (Figure 2) and sNPFR (Figure S2) indicated that these proteins were clustered with the Coleoptera group with *D. ponderosae*.

### 3.2. RT-qPCR

#### 3.2.1. Different Developmental Stages and Body Parts

**Figure 3:**
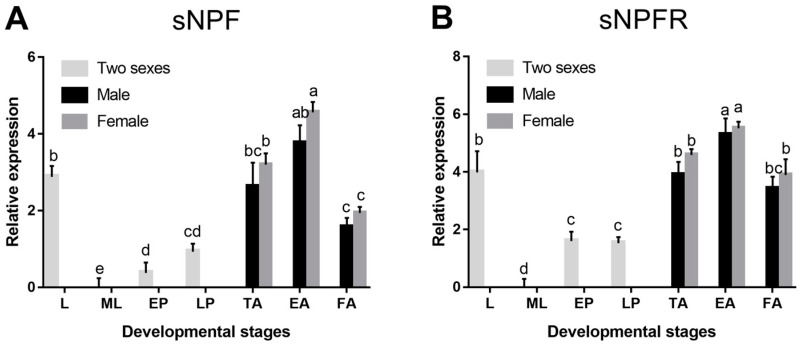
Relative mRNA expression levels of sNPF (**A**) and sNPFR (**B**) in different developmental stages of *D. armandi*. The relative expression levels were normalized with *β-actin* and *CYP4G55*. Different lowercase letters indicate significant differences at the 0.05 level (one-way ANOVA, Tukey test). All values are mean ± SE, *n* = 3. L, larvae; ML, mature larvae; EP, early stage pupae; LP, late stage pupae; TA, teneral adult; EA, emerged adult; FA, feeding adult.

sNPF and sNPFR were detected in all developmental stages of *D. armandi*. All of them were expressed at the highest level in emerged adults and the lowest in mature larvae (Figure 3A,B). However, statistically significant differences were not found between sexes in adults (*p* > 0.05).

**Figure 4:**
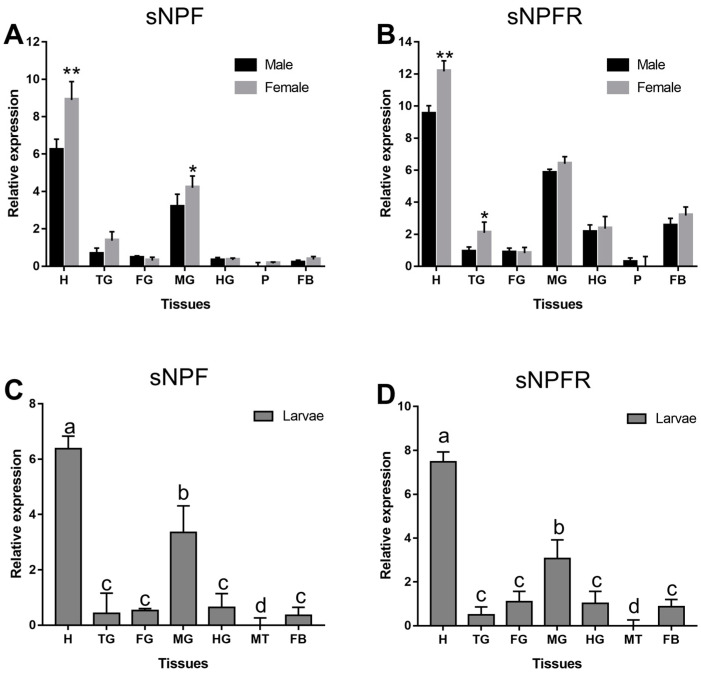
Relative expression levels of emerged adults of sNPF (**A**) and sNPFR (**B**), and larvae of sNPF (**C**) and sNPFR (**D**) in different body parts of *D. armandi*. The relative expression levels were normalized with *β-actin* and *CYP4G55*. Different lowercase letters indicate significant differences at the 0.05 level. The asterisk indicates a significant difference between female and male expression levels (* *p* < 0.05, ** *p <* 0.01, independent Student’s *t*-test). All values are mean ± SE, *n* = 3. H, head; TG, thoracic ganglion; FG, foregut; MG, midgut; HG, hindgut; P, pheromone gland; MT, Malpighian tubule; FB, fat body.

sNPF and sNPFR were expressed at varying levels in multiple body parts and with occasional sex differences (Figure 4). sNPF was highly expressed only in the head and midgut of adults (Figure 4A) and larvae (Figure 4C), while sNPFR of adults and larvae (Figure 4B,D) was expressed in different tissues except pheromone gland and Malpighian tubule. Moreover, all of them were the highest expressed in the head, followed by midgut in adults and larvae. sNPF was more highly expressed in female than male in the head (*p* < 0.01) and midgut (*p* < 0.05) (Figure 4A), while the expression of sNPFR in the head (*p* < 0.01) and thoracic ganglion (*p* < 0.05) of female was remarkably higher than that of male (Figure 4B).

#### 3.2.2. Starvation and Re-Feeding Assays

**Figure 5:**
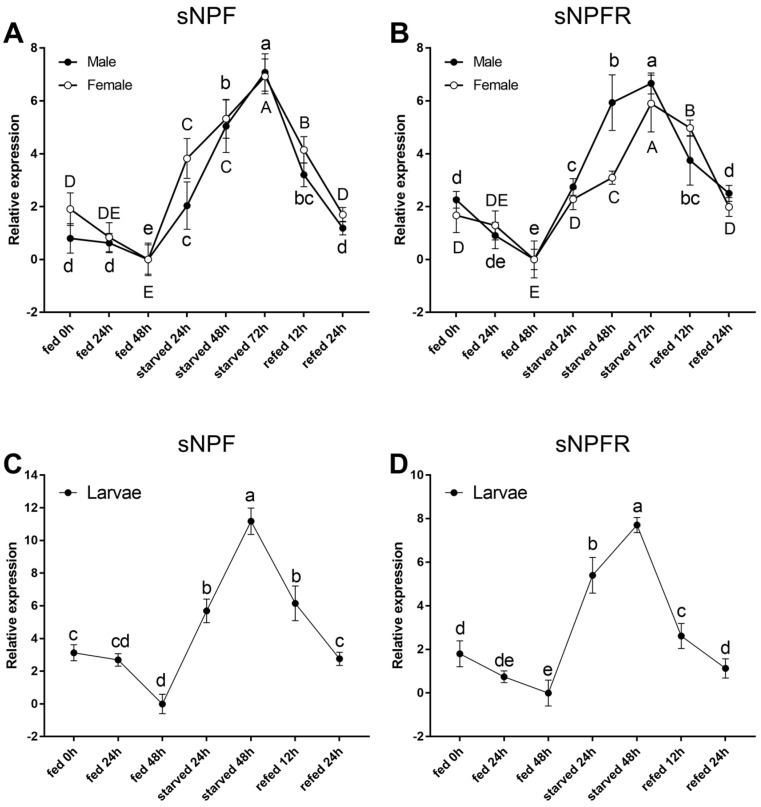
Relative expression levels of emerged adults of sNPF (**A**) and sNPFR (**B**), and larvae of sNPF (**C**) and sNPFR (**D**) in *D. armandi* after starvation and subsequent re-feeding treatment. The relative expression levels were normalized with *β-actin* and *CYP4G55*. Different letters indicate significant differences at the 0.05 level. Tukey tests: uppercase for males, lowercase for females, and uppercase for males and larvae; no letter means no significant difference among all times. All values are mean ± SE, *n* = 3.

The expression levels of both the sNPF and sNPFR transcripts showed a similar response to starvation stress (Figure 5). Compared with the fed groups, the sNPF and sNPFR expression levels in adults (*p* < 0.05, Figure 5A,B) and larvae (*p* < 0.05, Figure 5C,D) were significantly upregulated in the starved groups and reached the highest at 72 h. Furthermore, during the refeeding treatment after food deprivation, the sNPF and sNPFR expression levels decreased steadily and then recover the original level (*p* < 0.05, Figure 5).

### 3.3. Efficiency Analysis of RNAi on sNPF and sNPFR

#### 3.3.1. Effect of dsRNA Treatment on sNPF and sNPFR Transcript Level

**Figure 6:**
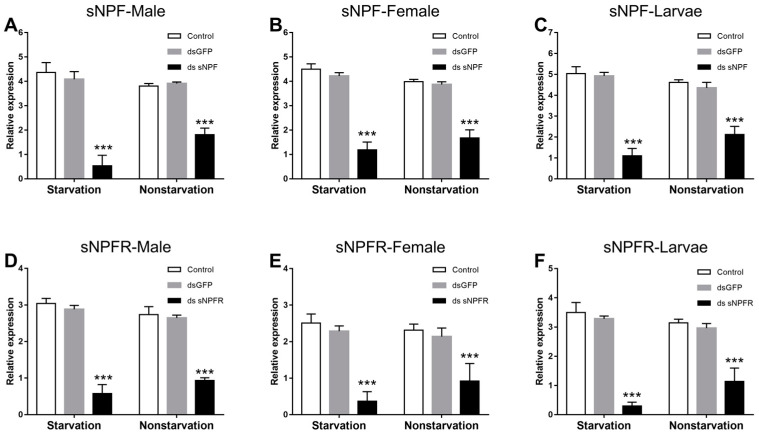
Knockdown of sNPF-Male (**A**), sNPF-Female (**B**), sNPF-Larvae (**C**), sNPFR-Male (**D**), sNPFR-Female (**E**), and sNPFR-Larvae (**F**) expression levels in *D. armandi* after dsRNA injection for 72 h in the states of starvation and non-starvation. The relative expression levels were normalized with *β-actin* and *CYP4G55*. The asterisk indicates a significant difference betweent dsRNA reatment group and the two control groups (*** *p* <0.001, one-way ANOVA). All values are mean ± SE, *n* = 3.

Compared with the two control groups, both in the starvation and the feeding groups, the expression level of sNPF (Figure 6A–C) and sNPFR (Figure 6D–F) in adults (*p* < 0.01) and larvae (*p* < 0.01) was significantly downregulated at 72 h after dsRNA injection, respectively, but the expression level of sNPF and sNPFR in the starvation group was reduced more than in the feeding group (*p* < 0.05).

#### 3.3.2. Effect of dsRNA Treatment on Mortality and Body Weight

**Figure 7:**
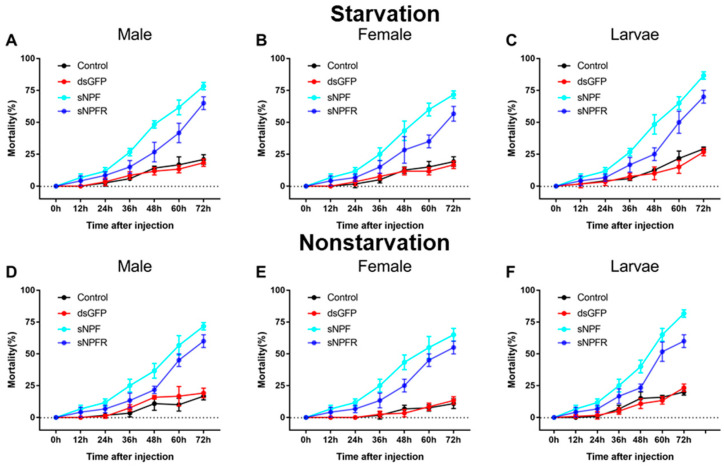
Mortality responses of RNAi *D. armandi* male (**A**), female (**B**), and larvae (**C**) in starvation and male (**D**), female (**E**), and larvae (**F**) in non-starvation groups at different time points after injection. Mortality responses of dsRNA-treated, dsGFP-injected, and non-injected in *D. armandi* to different time points (0, 12, 24, 36, 48, 60, and 72 h). All experiments were analyzed by Student’s *t*-test, and in (**A**–**F**) experimental beetles significantly differed (*p <* 0.01) from two controls.

Both in starvation and feeding groups, the mortality of the dsRNA-treated adults (*p* < 0.01) and larvae (*p* < 0.01) was higher than that of the dsGFP-injected and non-injected controls (Figure 7). When the adults and larvae were injected with dsRNA from 0 to 72 h, the mortality increased remarkably. The highest mortality was found at 72 h after injection of ds sNPF. Furthermore, the mortality of larvae was the highest and female adults was the lowest at 86.7% and 55%, respectively (Figure 7C,E). Additionally, the mortality in the starvation group was significantly higher than feeding group (*p* < 0.05, Figure 7).

**Figure 8:**
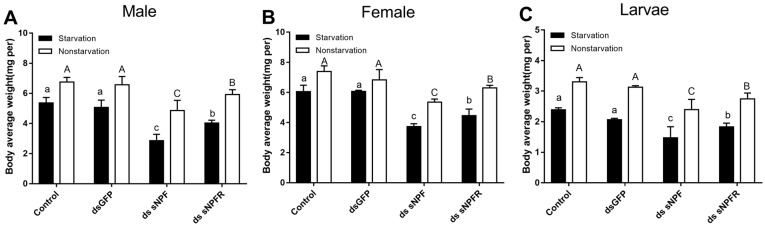
Effect of ds sNPF and ds sNPFR on body average weight. Samples were collected and assayed at 72 h after injection. In the RNAi experiments, body average weight of male (**A**), female (**B**), and larvae (**C**) in the states of starvation and non-starvation was analyzed. Different letters indicate significant differences at the 0.05 level. Tukey tests: uppercase for non-starvation, lowercase for starvation. All values are mean ± SE, *n* = 3.

Compared with the two control groups, the average weight of adults and larvae both in the starvation group and the feeding group decreased significantly after 72 h of injection of ds sNPF and ds sNPFR, and among them, in the starvation group, the male had the most weight loss, with 46.6%, and the female adults had the least weight loss, with 27.5% (Figure 8A,B), but we found more reductions in the dsNPF injection group (*p* < 0.05). The proportional change was about 15% in controls between the starvation and non-starvation states. In addition, the average weight of adults and larvae after dsRNA injection in the starvation group decreased more compared with the feeding group (*p* < 0.05, Figure 8). These results suggested that the RNAi-mediated downregulation of sNPF had an inhibitory effect on food intake in beetles.

#### 3.3.3. Effects of dsNPF on Regulating Energy Metabolism

**Figure 9:**
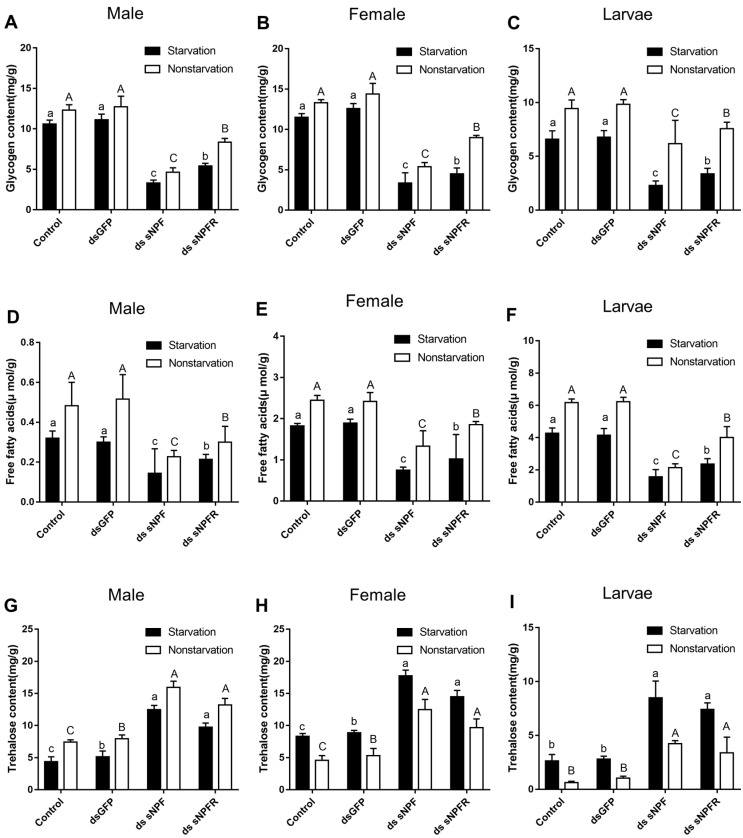
Effect of ds sNPF and ds sNPFR on energy metabolism. Samples were collected and assayed at 72 h after injection. Whole-body homogenates were used to measure glycogen of male (**A**), female (**B**), and larvae (**C**); free fatty acids of male (**D**), female (**E**), and larvae (**F**); and trehalose contents of male (**G**), female (**H**), and larvae (**I**) in the state of starvation and non-starvation. Different letters indicate significant differences at the 0.05 level. Tukey tests: uppercase for non-starvation, lowercase for starvation. All values are mean ± SE, *n* = 3.

The glycogen and free fatty acid contents of adults and larvae were remarkably reduced after injection of ds sNPF (*p* < 0.01) and ds sNPFR (*p* < 0.05) for 72 h compared with the two control groups (Figure 9). Both in the starvation group and the feeding group, the decrease was more obvious in the ds sNPF injection group (*p* < 0.05). More interestingly, the trehalose content of adults and larvae increased remarkably at 72 h after injection of ds sNPF (*p* < 0.01) and ds sNPFR (*p* < 0.05) compared with the two control groups. Moreover, the content of glycogen and free fatty acid decreased in the starvation group after injection of dsRNA compared with the feeding group. By contrast, the content of trehalose increased (Figure 9).

## 4. Discussion

In the present study, one sNPF-encoding cDNA was identified in *D. armandi*, which is in line with the results of most insect studies thus far. The cDNA sequence of sNPF confirmed the presence of 102 amino acids, and mature short neuropeptide F was predicted with an amidated RLRFamide C-terminus, which is almost identical with the C-terminal sequence RLRFamide of sNPF peptides in numerous arthropods [1]. We also cloned cDNAs encoding sNPFR from *D. armandi*, which code a length of 428 amino acids. It contained some characteristic residues in the seven transmembrane domains including GN in helix 1, NLX3-DX8P in helix 2, SX3LX2IX2DRY in helix 3, WX8P in helix 4, FX2PX7Y in helix 5, FX3WXP in helix 6, and NPX2YX6F in helix 7 [38]. This typical pattern indicated that the sNPFR belongs to the rhodopsin-like GPCR superfamily.

We found that both the sNPF and sNPFR transcripts were expressed throughout *D. armandi* development, suggesting that the sNPF signaling system may be involved in the regulation of some physiological and developmental processes of beetles. A similar result was observed in *B. dorsalis*; differential transcription profiles of sNPF and sNPFR were found in the different developmental stages [10]. In addition, the expression levels of these genes were also assessed in different life stages of *R. padi* [13]. In this study, sNPF and sNPFR showed high transcription levels in larvae and adults, which could be because they had sensitive responses to host volatiles [21]. Moreover, sNPF and sNPFR were more highly expressed in female than male in the head. Female adults usually occur in adverse environmental conditions when they need to search for new host plants. Additionally, females are more important in the location and detoxification of host monoterpenes than male adults [28].

As indicated by qPCR, the sNPF and sNPFR transcripts were mainly detected in the central nervous system of *B. dorsalis* [10]. The distribution of the sNPF transcript seems to be limited to nervous tissues in *S. gregaria* [20]. Furthermore, both mass spectrometry and RT-PCR analyses of *B. mori* revealed that sNPF was localized in the tissues of neural origin [18]. Immunolabeled structures with sNPF were observed in the brain and ventral nerve cord VNC of burying beetles, *Nicrophorus vespilloides* [39]. Additionally, sNPF was detected in abdominal segmental nerves of the large pine weevil, *Hylobius abietis,* by mass spectrometry, and it contained the axons of neurosecretory neurons in abdominal ganglia [40]. However, it has been reported that sNPFs of *D. melanogaster* were also expressed in the midgut in addition to the CNS [41]. This is consistent with the situation in *D. armandi.* A high expression of sNPFR was also observed in the CNS of *S. gregaria* and *B. dorsalis* [10,20]. However, sNPFR expression was observed in several other tissues, in addition to the nervous system, such as the gut, ovaries, and fat body of *Solenopsis invicta* and *D. melanogaster* [42,43]. Furthermore, the expression of sNPFR appeared to be the highest in the brain of *T. molitor*, but it was also present in the ejaculatory duct. The distribution of the sNPF receptor of *D. armandi* is in accordance with these insect species.

In this study, we observed that the expression level of sNPF in *D. armandi* increased with the prolongation of starvation time, but the following refeeding experiments caused a continuous drop and returned to the original level. These results were similar to other insect species reported previously. In *B. dorsalis*, the sNPF and sNPFR expression levels revealed significant starvation-induced patterns [10]. The expressions of sNPF and sNPFR were also upregulated after food deprivation in the brain of *A. mellifera* [14]. Although sNPF has been widely regulated in feeding behavior, opposing functions have also been reported in *S. gregari*a; transcript levels of sNPF and sNPFR were lower in starved animals compared to normally fed controls [20,43]. Meanwhile, the expression of sNPFR showed a reduction after starvation in the mollusk, *Crassostrea gigas* [44]. Additionally, sNPFR expression levels decreased in the central nervous system amongst other tissues during food deprivation in *B. mori* [18].

To further study the function of the sNPF signaling pathway in beetle feeding behavior, we knocked sNPF down in adults and larvae using RNAi technology. The results indicated it could effectively suppress the expression of sNPF. In *D. melanogaster,* overexpression of sNPFs regulated body size and promoted food intake [12]. Additionally, silencing the sNPF of *R. padi* significantly decreased food uptake by weighing wheat leaves [13]. However, injection of either of the two sNPF isoforms strongly decreased food intake and silencing sNPF significantly stimulated food intake in *S. gregaria* [20,45]. Specifically, the beetles had a smaller body weight after sNPF knockdown. This maybe led beetles to eat less, resulting in a delay of development and growth, and causing a reduction in body weight. It indicates that silencing sNPF in *D. armandi* inhibits their appetite, likely resulting in a change of food intake, which suggests that sNPF may be a key factor to control feeding behavior.

The mortality was significantly higher in adults and larvae after injecting with ds*RNA* than that of two controls. Therefore, sNPF and sNPFR silencing not only suppresses their expression levels but also leads to an increase of mortality. These results were in consonance with previous studies in *R. padi,* wherein injection with dssNPF led to a high mortality of this species when undergoing prolonged starvation [13].

A previous study on *S. gregaria* reported that the sNPF signaling system is regulated by the components of the insulin/IGF signaling pathway [46]. In this study, we found that the *s*NPF of *D. armandi* regulated feeding behavior by affecting energy metabolism, in which sNPF knockdown resulted in an increase in trehalose and a decrease in glycogen. Presumably, the sNPF signaling system not only promotes energy storage but also suppresses energy utilization with less food intake or even starvation. In addition, feeding behavior provides more nutrients and reduces the metabolic requirements of stored glycogen or free fatty acid. This pattern is in line observations of *D. melanogaster* [7]. Silencing the sNPF in the DLPs of fruit flies significantly increased hemolymph levels of glucose and trehalose compared to controls. Furthermore, the Crustacean cardioactive peptide (CCAP) RNAi in larvae of *L. decemlineata* had a higher mRNA level of sNPF, consumed minimal foliage, grew slowly, and possessed a small body size. Moreover, silencing *ccap* lowered trehalose content [47]. This suggests that the sNPF of *D. armandi* is also involved in the insulin or CCAP signal system to regulate energy metabolism.

It is worth noting that the regulation of feeding behavior is not only involved in sNPF, but also related to the expression changes of sNPF receptor in invertebrates. In this study, we found that function on the sNPFR knockdown was in consonance with that of sNPF. These results indicate sNPFR has similar effect on regulating food intake. Nevertheless, it is unknown as to how the sNPF/sNPFR signaling system is regulated in order to control feeding behavior at the molecular, cellular, and neural circuit levels in *D. armandi*. The specific mechanism will need to be elucidated by further investigation and study.

## Figures and Tables

**Table 1:** Physicochemical properties and cellular localization of putative D. armandi sNPF and sNPFR proteins.

Gene Name	Accession No.	ORF Size (aa/bp) ^a^	MW (KDa) ^a^	IP ^a^	Signal Peptide Prediction ^b^
sNPF	MW174224	102/309	11.48	8.06	SP 0.957 mTP 0.027 other 0.066
sNPFR	MW174225	428/1287	49.27	8.20	SP 0.039 mTP 0.680 other 0.703

Note: ORF, open reading frame; MW, molecular weight; IP, isoelectric point; SP, secretory pathway signal peptide; mTP, mitochondrial targeting peptide. ^a^ As predicted by the PROTPARAM program. ^b^ As predicted by TARGET P1.1.

**Table 2:** Amino acid identity of sNPF and sNPFR genes isolated from D. armandi with related gene sequences in other insects.

Gene	BLAST Matches in GenBank			
Name	Species	Name	Accession Number	% Identity ^a^
sNPF	*Dendroctonus ponderosae*	short neuropeptide F-like	XP_019767920.1	96
	*Sitophilus oryzae*	short neuropeptide F-like	XP_030755875.1	67
	*Rhynchophorus ferrugineus*	short neuropeptide F-like	QGA72576.1	60
sNPFR	*Dendroctonus ponderosae*	prolactin-releasing peptide receptor-like	XP_019761868.1	97
	*Rhynchophorus ferrugineus*	prolactin-releasing peptide receptor-like	QGA72517.1	82
	*Anopheles gambiae*	short neuropeptide F receptor	ABD96049.1	66

Note: ^a^ As predicted by BLAST (http://www.ncbi.nlm.nih.gov, accessed on 12 September 2020).

## Data Availability

Data is contained within this article and the Supplementary Materials.

## Supplementary Materials

The following are available online at https://www.mdpi.com/article/10.3390/insects12090844/s1. Table S1: Primer sequences used in the research. Figure S1: Comparison of the amino acid sequence of D. armandi sNPFR with those of other species. They include *Dendroctonus ponderosae* (Dp sNPFR, XP_019761868.1), *Rhynchophorus ferrugineus* (Rf sNPFR, QGA72517.1), *Sitophilus oryzae* (So sNPFR, XP_030746843.1), *Diabrotica virgifera virgifera* (Dv sNPFR, XP_028151444.1), *Leptinotarsa decemlineata* (Ld sNPFR, XP_023019356.1), *Anoplophora glabripennis* (Ag sNPFR, XP_018571523.1), *Aethina tumida* (At sNPFR, XP_019867538.1). The predicted seven transmembrane domains are underlined by a solid line. Identical amino acid residues in all proteins are shown in black,grey parts indicate similar amino acids. Figure S2: Phylogenetic trees based on amino acid sequences of sNPFR from other insect species. They include *Leptinotarsa decemlineata* (XP_023019356.1), *Diabrotica virgifera virgifera* (XP_028151444.1), *Anoplophora glabripennis* (XP_018571523.1), *Aethina tumida* (XP_019867538.1), *Tribolium castaneum* (XP_015833194.1), *Nicrophorus vespilloides* (XP_017779804.1), *Photinus pyralis* (XP_031348170.1), *Onthophagus taurus* (XP_022909330.1), *Dendroctonus ponderosae* (XP_019761868.1), *Rhynchophorus ferrugineus* (QGA72517.1), *Sitophilus oryzae* (XP_030746843.1), *Bicyclus anynana* (XP_023934255.1), *Aphantopus hyperantus* (XP_034831358.1), *Helicoverpa armigera* (XP_021184191.1), *Bombyx mori* (ALP48446.1), *Ostrinia furnacalis* (XP_028173698.1), *Chilo suppressalis* (ALM88307.1), *Culex quinquefasciatus* (AVR59280.1), *Aedes aegypti* (XP_021705044.1), *Anopheles sinensis* (KFB49968.1), *Anopheles gambiae* (ABD96049.1). The phylogenetic tree constructed by the Maximum Likelihood method using the amino acidic substitution model WAG++G+I+F122 in MEGA 6.0. Bootstrap values after 500 pseudo-replicates are shown at nodes.The bootstrap values (in %) are given at each branch point. The black dot indicates D. armandi sNPFR.
