# Supplementary material for: Identification of the Short Neuropeptide F and Short Neuropeptide F Receptor Genes and Their Roles of Food Intake in Dendroctonus armandi
Source: Insects. 2021 Sep 19;12(9):844. doi: 10.3390/insects12090844 (PMC8469826; doi:10.3390/insects12090844)
Supplement: Supplementary file 1 [file insects-12-00844-s001.zip › insects-1318525-supplementary.pdf]

Supplementary Information

Table S1. Primer sequences used in the research.

**Gene**	Sequence （5′ → 3′）		purpose
	Forward	Reverse	
**sNPF**	ACCTCAGCTCCCTCGTAT	ACTCCTTGCGGAAGTTGTCG	cDNA
	ACAACGCAATGAAAGTATTCTCTCT	AATCGGTTATCACACAGTTTCAAG	3′RACE
	TCGCCAGAATACGAGGGAGC	TAATTGTGGTGACCGTCCAG	5′ RACE
	AACAACGCAATGAAAGTATTCTCTC	GGGATACGTTTAGTTGTCGTTAGTG	Full-length
	TTCAACTCTTAACACCCAGTCTTCG	TGAATAAGCATGTCAATCAGTTCTC	qPCR
	TAATACGACTCACTATAGGGTCAGCCCCCTCTTATTCTGG	TAATACGACTCACTATAGGGGTGATGTCATTCAAGCCGGT	RNAi
**sNPFR**	ACGAATCGCATGCTGATCG	TCCTTGCGGAAGTTGTCG	cDNA
	GGCGGTGTTTCTCATCTCCT	TGGCTTAACGACAACTTCCG	3′RACE
	GTGAAGGGCACAGCCAATAC	CATCAAGGTAAGCGTGGAAA	5′ RACE
	GCTGACCGAAGCAATCAACGTGACC	GAACTAGGGGTAGTCTTGTGGATGT	Full-length
	AGCAGTGGTATCAACGCAGAG	GCATTCATTGGCATCATCAG	qPCR
	TAATACGACTCACTATAGGGACATTTCCACGCTTACCTTG	TAATACGACTCACTATAGGGTCCCTATCCGCTTCCTCTTT	RNAi
**GFP**	TAATACGACTCACTATAGGGATGGTGTTCAATGCTTTTCA	TAATACGACTCACTATAGGGCTCTCTTTTCGTTGGGGTCT	RNAi

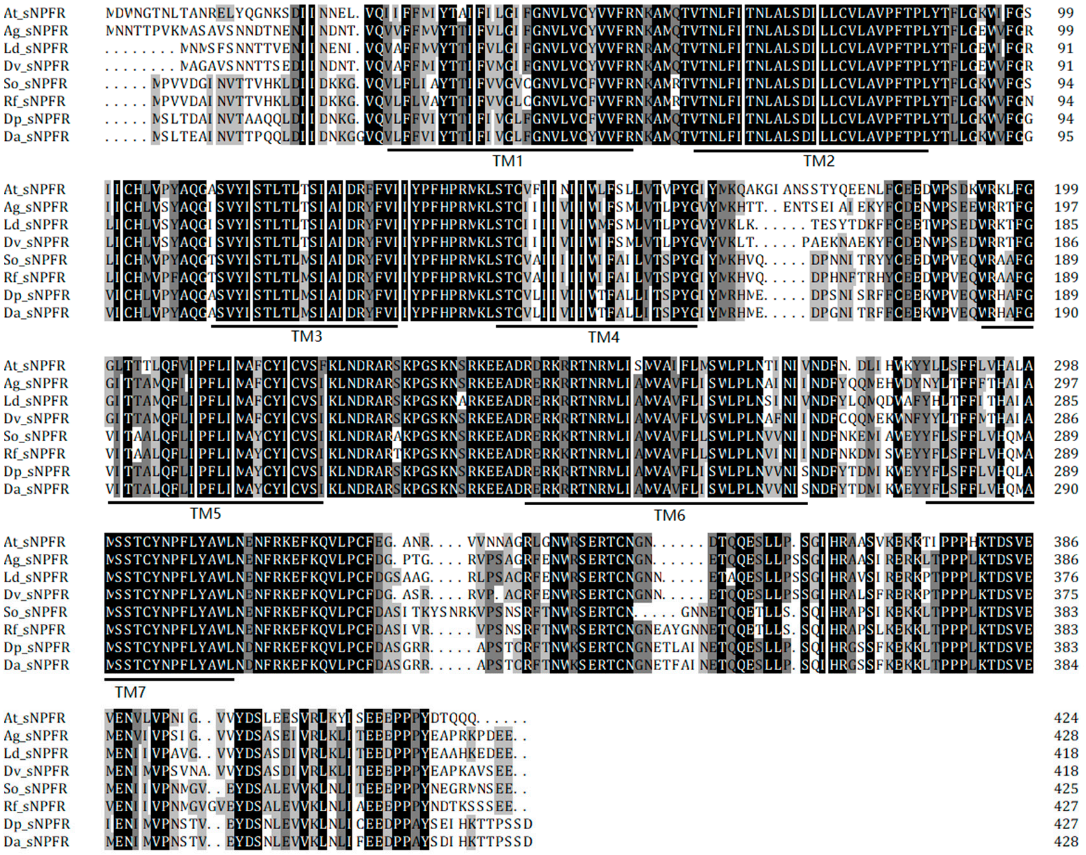

Figure S1. Comparison of the amino acid sequence of *D. armandi* sNPFR with those of other species. They include *Dendroctonus ponderosae* (Dp sNPFR, XP_019761868.1), *Rhynchophorus ferrugineus* (Rf sNPFR, QGA72517.1), *Sitophilus oryzae* (So sNPFR, XP_030746843.1), *Diabrotica virgifera virgifera* (Dv sNPFR, XP_028151444.1), *Leptinotarsa decemlineata* (Ld sNPFR, XP_023019356.1), *Anoplophora glabripennis* (Ag sNPFR, XP_018571523.1), *Aethina tumida* (At sNPFR, XP_019867538.1). The predicted seven transmembrane domains are underlined by a solid line. Identical amino acid residues in all proteins are shown in black,grey parts indicate similar amino acids.

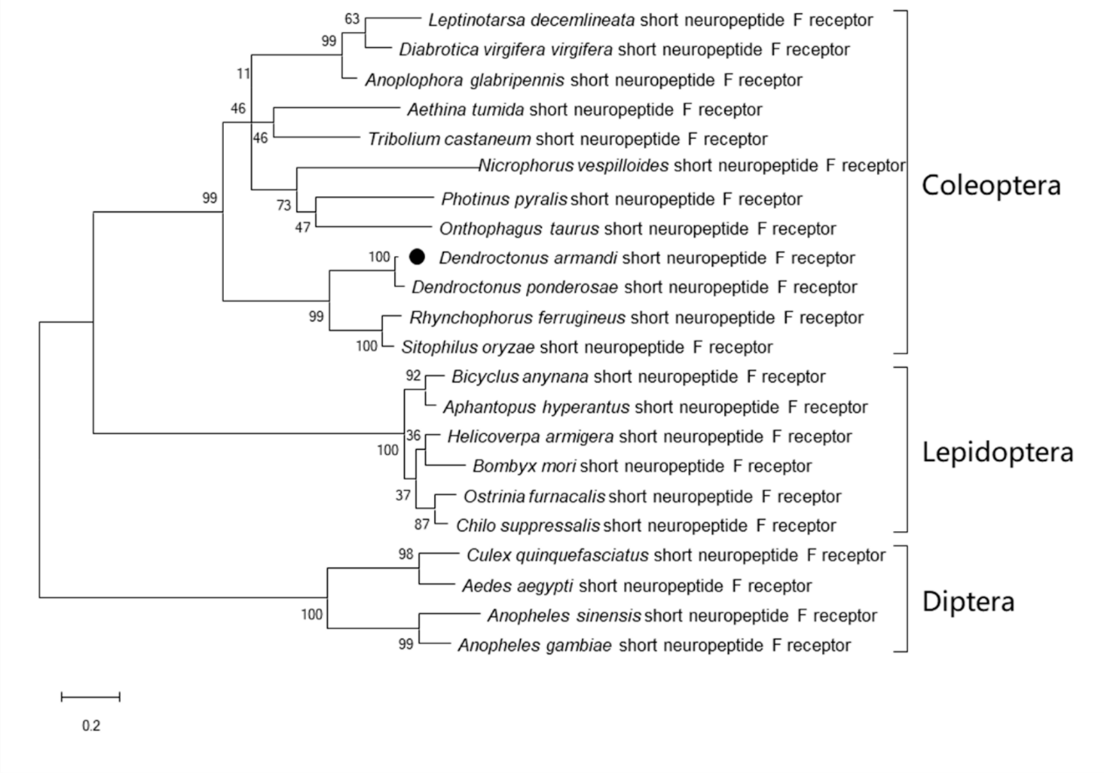

Figure S2. Phylogenetic trees based on amino acid sequences of sNPFR from other insect species. They include *Leptinotarsa decemlineata* (XP_023019356.1), *Diabrotica virgifera virgifera* (XP_028151444.1), *Anoplophora glabripennis* (XP_018571523.1), *Aethina tumida* (XP_019867538.1), *Tribolium castaneum* (XP_015833194.1), *Nicrophorus vespilloides* (XP_017779804.1), *Photinus pyralis* (XP_031348170.1), *Onthophagus taurus* (XP_022909330.1), *Dendroctonus ponderosae* (XP_019761868.1), *Rhynchophorus ferrugineus* (QGA72517.1), *Sitophilus oryzae* (XP_030746843.1), *Bicyclus anynana* (XP_023934255.1), *Aphantopus hyperantus* (XP_034831358.1), *Helicoverpa armigera* (XP_021184191.1), *Bombyx mori* (ALP48446.1), *Ostrinia furnacalis* (XP_028173698.1), *Chilo suppressalis* (ALM88307.1), *Culex quinquefasciatus* (AVR59280.1), *Aedes aegypti* (XP_021705044.1), *Anopheles sinensis* (KFB49968.1), *Anopheles gambiae* (ABD96049.1). The phylogenetic tree constructed by the Maximum Likelihood method using the amino acidic substitution model WAG++G+I+F122 in MEGA 6.0. Bootstrap values after 500 pseudo-replicates are shown at nodes.The bootstrap values (in %) are given at each branch point. The black dot indicates *D. armandi* sNPFR.
